# Peritumoral Brain Edema in Meningiomas May Be Related to Glymphatic Dysfunction

**DOI:** 10.3389/fnins.2021.674898

**Published:** 2021-04-22

**Authors:** Cheng Hong Toh, Tiing Yee Siow, Mauricio Castillo

**Affiliations:** ^1^Department of Medical Imaging and Intervention, Chang Gung Memorial Hospital at Linkou, Taoyuan, Taiwan; ^2^College of Medicine, Chang Gung University, Taoyuan, Taiwan; ^3^Department of Radiology, University of North Carolina School of Medicine, Chapel Hill, NC, United States

**Keywords:** meningioma, peritumoral brain edema, glymphatic system, diffusion-tensor imaging, brain interstitial fluid

## Abstract

The pathogenesis of peritumoral brain edema (PTBE) in meningiomas remains unclear. The glymphatic system is recently recognized as a pathway for waste clearance and maintaining fluid balance in the brain parenchymal interstitium. We aimed to investigate if the PTBE volume of meningiomas correlates with their glymphatic function. A total of 80 meningioma patients (mean age, 58.8 years; 37 men) and 44 normal subjects (mean age 53.3 years; 23 men) who had preoperative diffusion-tensor imaging for calculation of the analysis along the perivascular space (ALPS) index were retrospectively included. Information collected from each patient included sex, age, tumor grade, Ki-67 index, tumor location, tumor volume, PTBE volume and ALPS index. Comparisons of ALPS index among meningiomas without PTBE, meningiomas with PTBE, and normal subjects were performed using analysis of covariance with Bonferroni correction and adjustments for age and sex. Pearson correlation coefficient and multivariable linear regression analyses were performed to identify factors associated with PTBE volume. Group comparisons revealed that the ALPS index was significantly higher (*P* < 0.05) in meningiomas without PTBE vs. meningiomas with PTBE and normal subjects. On the other hand, ALPS index was not different between meningiomas with PTBE and normal subjects. On Pearson correlation and multivariable linear regression analyses, the ALPS index was the only factor significantly (*P* < 0.05) associated with PTBE volume. In conclusion, PTBE volume inversely correlated with ALPS index in meningiomas. PTBE formation in meningiomas may be related to glymphatic dysfunction.

## Introduction

Meningiomas account for 37.6% of primary brain tumors in population-based studies (Ostrom et al., 2019). Up to 67% of meningiomas have peritumoral brain edema (PTBE) (Hou et al., 2013). PTBE increases intracranial pressure, complicates surgical resection, and is associated with higher risk of seizures (Simis et al., 2008), postoperative intracranial hemorrhages (Sindou and Alaywan, 1998) and recurrences (Mantle et al., 1999; Simis et al., 2008). Pathogenesis of PTBE remains unclear and there are conflicting reports on correlations between PTBE extension and imaging features such as tumor size or volume (Bitzer et al., 1997; Tamiya et al., 2001; Paek et al., 2002; Lee et al., 2008; Simis et al., 2008; Kim et al., 2011), tumor-brain interface (Tamiya et al., 2001; Nakano et al., 2002; Simis et al., 2008; Kim et al., 2011; Sapkota et al., 2020), tumor location (Osawa et al., 2013; Sapkota et al., 2020), venous outflow obstruction (Kim et al., 2019), tumor blood supply pattern (Tamiya et al., 2001; Osawa et al., 2013), and tumor T2-weighted signal (Tamiya et al., 2001; Nakano et al., 2002; Kim et al., 2011; Sapkota et al., 2020).

The glymphatic system is recently recognized as a pathway for waste clearance and maintaining fluid balance in the brain parenchymal interstitium (Rasmussen et al., 2018). CSF from the subarachnoid space flows into brain parenchyma through periarterial spaces of the penetrating arteries under the influence of Aquaporin-4 water channels and mixes with parenchymal interstitial fluid. The interstitial fluid moves with its solutes toward the perivenous and perineuronal spaces, where it leaves brain parenchyma. From there, CSF exits the intracranial compartment via the perineural sheaths of some cranial and spinal nerves, dural lymphatics, and arachnoid granulations. Glymphatic failure has been proposed as a final pathway to dementia (Nedergaard and Goldman, 2020) and is responsible for early tissue swelling and edema after ischemic infarct (Mestre et al., 2020). In glioma-bearing mice, CSF flow to extracranial spaces is reduced (Ma et al., 2019b) but in extra-axial tumors such as meningiomas, alterations of glymphatic function related to PTBE remain unclear.

In the diffusion tensor image analysis along the perivascular space (DTI-ALPS) method (Taoka et al., 2017), ALPS index, a diffusion metric that estimates the diffusivity along the perivascular spaces of medullary veins, has been used to assess glymphatic function. Alterations of the ALPS index correlate with the Mini-Mental State Examination score of dementia patients and is thought to reflect glymphatic dysfunction (Taoka et al., 2017). ALPS index is also significantly lower in normal pressure hydrocephalus patients and suggests glymphatic dysfunction as evidenced by delayed clearance of intrathecally injected gadobutrol (Ringstad et al., 2017; Eide and Ringstad, 2019). Therefore, the ALPS index may serve as a surrogate marker for identifying altered glymphatic function and quantifying glymphatic activity.

To the best of our knowledge, the relationship between the function of the glymphatic system and PTBE of meningiomas has not been investigated. The DTI-ALPS method may help understand the role of the glymphatic system in PTBE formation and may aid in the development of treatments for reducing PTBE preoperatively. We hypothesized that PTBE of meningiomas is associated with dysfunction of glymphatic system as evidenced by an altered ALPS index.

## Materials and Methods

### Study Subjects

Approval for reviewing the patients’ clinical data and preoperative MRI findings was obtained from our Institutional Review Board. Between 2006 and 2017, a total of 90 consecutive patients with a histopathological diagnosis of meningioma underwent preoperative DTI at our institution. A total of 10 patients were excluded due to motion artifacts (*n* = 2), bihemispheric tumors (*n* = 4), infratentorial tumors (*n* = 2) and *trans-*arterial tumor embolization before MRI examinations (*n* = 4). Thus, a total of 80 patients (43 women, 37 men; mean age, 58.8 ± 13.5 years; age range, 24–90 years) were analyzed. No patients had begun corticosteroid treatment, radiation therapy, chemotherapy or had previous brain surgery at the time of their MRI studies. An overview of patient characteristics is found in Table 1. As healthy controls, 44 subjects (21 women, 23 men; mean age 53.3 ± 10.0 years; age range, 27–83 years) with normal brain MRI examinations were also included.

**TABLE 1:** Patient characteristics.

Characteristics	All meningioma
No. of patients	80
Mean age ± SD (y)	58.8 ± 13.5
**Sex**	
Man	37
Woman	43
**Tumor location**	
Sphenoid ridge	12
Parasellar	1
Frontal base	2
Convexity	39
Parasagittal	23
Intraventricular	2
Tentorium	1
**Tumor grade**	
Grade I	61
Grade II	15
Grade III	4
**Tumor volume (cm^3^)**	
1–8	7
8–27	15
27–64	28
64–125	22
>125	8
**Edema volume (cm^3^)**	
0	24
1–27	15
27–64	15
64–125	17
>125	9

*Except where indicated, data are numbers of patients. SD = standard deviation.*

### Clinical and Imaging Information

Patient medical records and MRI studies were retrospectively reviewed to collect clinical and imaging information including sex, age, tumor grade (grade I or grade II/III), tumor proliferation index Ki-67, tumor location, and the side of the cerebral hemisphere or posterior fossa in which the tumor was located (right or left). Histopathologic diagnosis was made by a board-certified neuropathologist with 20 years of experience. Tumors were graded according to the 2016 World Health Organization classification. Tumor locations were skull base (frontal base, tuberculum sellae, parasellar, sphenoid ridge) and non-skull base (other locations).

### MRI

All MRI studies were performed using a 3T unit (Magnetom Tim Trio, Siemens, Erlangen, Germany) with a 12-channel phased-array head coil. All examinations included a T2-weighted, DTI, and T1-weighted sequences acquired in transverse plane before and after administration of 0.1 mmol/kg body weight gadopentetate dimeglumine (Magnevist; Schering, Berlin, Germany).

Diffusion tensor image was performed using a single-shot EPI with the following parameters: TR msec/TE msec, 5800/83; diffusion gradient encoding in 20 directions; *b* = 0, 1000 s/mm^2^; FOV, 256 × 256 mm; matrix size, 128 × 128; section thickness, 2 mm; and number of signals acquired, 4. A total of 50–60 sections without intersection gap were used to cover the cerebral hemispheres, brainstem and cerebellum. Generalized autocalibrating partially parallel acquisitions (GRAPPA) with reduction factor set at two were used during DTI acquisitions. Contrast-enhanced T1-weighted images (TR/TE, 2000/2.63 ms; section thickness, 1 mm; TI, 900 ms; acquisition matrix, 224 × 256 and FOV, 224 × 256 mm) were acquired after completion of the DTI sequence.

### Measurements of Tumor and PTBE Volume

**FIGURE 1:**
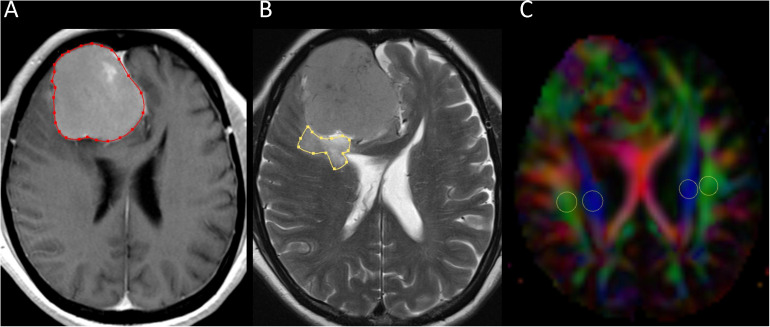
Example of how regions of interest (ROIs) were segmented. Transverse contrast-enhanced T1-weighted **(A)** and T2-weighted **(B)** images show manually drawn ROIs for measurement of meningioma and peritumoral brain edema volumes. Directionally encoded color map **(C)** illustrates ROIs of projection (blue area) and association (green area) fibers in bilateral periventricular regions.

Two neuroradiologists (with 16 and 6 years of experience) independently measured tumor and PTBE volumes of meningiomas using the software nordicICE (nordic Image Control and Evaluation Version 2, Nordic Imaging Lab, Bergen, Norway). A polygonal region-of-interest (ROI) was manually drawn to include the entire tumor on each transverse contrast-enhanced T1-weighted image. The ROIs on all images were then combined to form a whole tumor volume-of-interest for calculations of tumor volume. If multiple tumors were present, all of them were measured and their volumes summed up. The same method was used for the calculation of PTBE volume on transverse T2-weighted images. All ROIs did not include dural tails, areas of necrosis, or presumed calcification. An example of ROI segmentation is shown in Figure 1.

### Diffusion Tensor Image Analysis Along the Periventricular Space (DTI-ALPS)

Diffusion tensor image analysis along the perivascular space method (Taoka et al., 2017) was used to evaluate the glymphatic functions. This method evaluates the diffusivity along the perivascular space on a transverse slice at the level of lateral ventricle body. The medullary veins, accompanied by the perivascular spaces run perpendicular to the ventricular walls at the level of the lateral ventricular body in a right-left or left-right direction (i.e., *x*-axis in image coordinate). In this level, the corticofugal corona radiata projection fibers run in the craniocaudal direction (i.e., *z*-axis in image coordinate) adjacent to the lateral ventricles. The superior longitudinal fascicle, which represents the association fibers runs in the anterior-posterior direction (i.e., *y*-axis in image coordinate) and is located lateral to the corona radiata. As the perivascular space is nearly perpendicular to both the projection fibers and association fibers, the major differences between *x*-axis diffusivity in both fibers (*D*_xproj_ and *D*_xassoc_ for *x*-axis diffusivity in projection fiber and association fiber, respectively) and the diffusivity that is perpendicular to the *x*-axis and to the direction of fiber tracts (*y*-axis for projection fiber, where the diffusivity denoted as D_yproj_; *z*-axis for association fiber, where the diffusivity denoted as D_zassoc_) is the existence of perivascular space. To quantify glymphatic activity, ALPS index is defined as follows:

(1) $$\mathrm{ALPS}\,\mathrm{index}=\frac{\text{mean}\,\left(D_{x\,p\,r\,o\,j},D_{x\,a\,s\,s\,o\,c}\right)}{\text{mean}\,\left(D_{y\,p\,r\,o\,j},D_{z\,a\,s\,s\,o\,c}\right)}$$

Diffusion metric images were generated by using 3D Slicer version 4.10.2^1^. Two neuroradiologists independently drew the ROIs of bilateral projection (mean size, 29 ± 17 mm^2^) and association fibers (mean size, 26 ± 17 mm^2^) on a slice at the level of the lateral ventricular body based on directionality encoded map. ALPS index was computed according to the equation above (1). An example of ROI placement for ALPS index measurement is shown in Figure 1.

### Statistical Analysis

A commercially available statistical software package (SPSS 22; IBM, Armonk, NY, United States) was used for analysis, and *P* values < 0.05 were considered to indicate a statistical significance. Continuous variables are denoted as mean ± standard deviation, unless otherwise noted. The Kolmogorov-Smirnov test was used to assess the normality of continuous variables and guide the selection of a parametric or nonparametric test for comparison of variables.

The interobserver variability in the measurements of PTBE volume, tumor volume and ALPS index was assessed by intraclass correlation coefficients (ICCs) with 95% confidence intervals (CIs) based on an absolute-agreement, two-way random-effects model. The final values of PTBE volume, tumor volume, and ALPS index were obtained by taking the mean of two observers’ independent measurements.

Because there are no reference ranges for human ALPS index, potential interhemispheric, sex, and age differences in ALPS index were taken into account when comparing between normal subjects and meningioma patients. As such, comparisons of normal subjects with patients having right-side and left-side meningiomas were performed separately. Differences in bilateral ALPS indices among meningiomas without PTBE, meningiomas with PTBE, and normal subjects were assessed using analysis of covariance with Bonferroni correction and adjustments for age and sex.

In the group of meningiomas with PTBE, correlations of PTBE volume with age, sex, tumor grade, Ki-67, tumor location, tumor volume, and ALPS index were analyzed with the Pearson correlation coefficient. Variance inflation factors were used to detect multicollinearity. All variables were entered as potential covariates in the stepwise multivariable linear regression analysis to assess their associations with PTBE volume.

## Results

Among 80 patients, 44 had right-side meningiomas and 36 had left-side meningiomas. A total of 15 meningiomas were in deep locations (12 sphenoid ridge, 1 parasellar, and 2 frontal base). Other tumor locations included the convexities (*n* = 39), parasagittal (*n* = 23), tentorial (*n* = 1), and intraventricular (*n* = 2). Tumor grades were grade I in 61 patients, grade II in 15, and grade III in 4. Mean Ki-67 (%) was 4.8 ± 3.1 (range, 1 to 12).

There were excellent interobserver agreements in the measurements of meningioma volumes (ICC = 0.997, 95% CI = 0.995–0.998, *P* < 0.001), PTBE volumes (ICC = 0.996, 95% CI = 0.994–0.997, *P* < 0.001), right ALPS index (ICC = 0.960, 95% CI = 0.884–0.950, *P* < 0.001) and left ALPS index (ICC = 0.934, 95% CI = 0.900–0.57, *P* < 0.001). Mean meningioma volume (cm^3^) of 80 patients of was 58.6 ± 45.3 (range, 3.1–202.9). PTBE was present in 56 patients, with a mean volume (cm^3^) of 67.1 ± 46.8 (range, 4.1 to 171.3). PTBE was absent in 24 (30%) patients.

**FIGURE 2:**
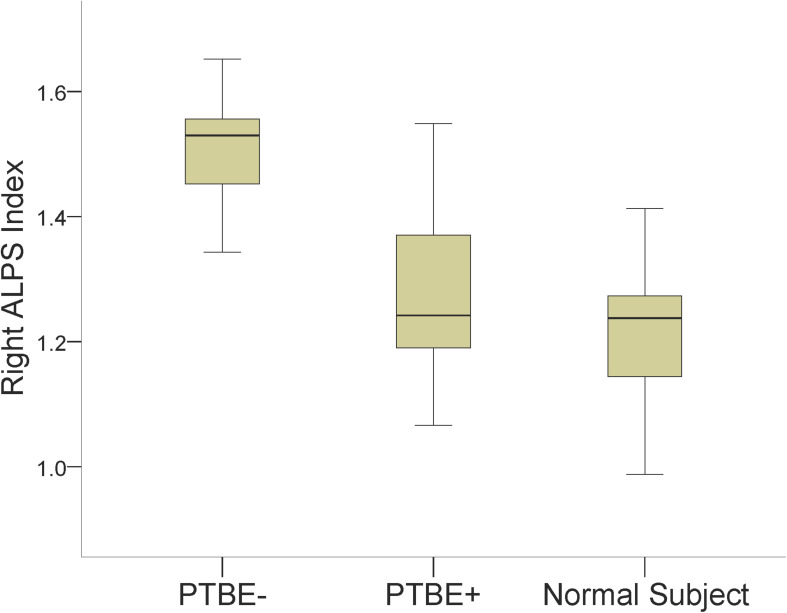
Boxplots show differences in the right ALPS index among right-side meningiomas without peritumoral brain edema (PTBE-), right-side meningiomas with peritumoral brain edema (PTBE+) and normal subjects.

Results of comparing the ALPS index among meningiomas without PTBE, meningiomas with PTBE and normal subjects are summarized in Table 2. The right ALPS index of right-side meningiomas without PTBE, right-side meningiomas with PTBE, and normal subjects was 1.502 ± 0.089, 1.260 ± 0.112, and 1.215 ± 0.093, respectively. The right ALPS index of meningiomas without PTBE was significantly higher than that of meningiomas with PTBE (*P* < 0.001) and normal subjects (*P* < 0.001). The right ALPS index was not significantly different between meningiomas with PTBE and normal subjects (*P* = 0.060). The contralateral (left) ALPS index of the three groups was not statistically significant (*P* = 0.148). Figure 2 shows the differences in right ALPS index among the three groups.

**TABLE 2:** Comparisons of ALPS index among meningiomas without PTBE, meningiomas with PTBE and normal subjects.

Index	Right-side meningioma		Normal subject (*n* = 44)	PTBE− vs. PTBE+		PTBE− vs. normal subject		PTBE+ vs. normal subject	
	PTBE− (*n* = 13)	PTBE+ (*n* = 31)		*P*-value	95% CI	*P*-value	95% CI	*P*-value	95% CI
Right ALPS	1.502 ± 0.089	1.260 ± 0.112	1.215 ± 0.093	<0.001	0.140, 0.304	<0.001	0.204, 0.357	0.060	−0.002, 0.118
Left ALPS	1.353 ± 0.213	1.263 ± 0.173	1.273 ± 0.105	0.615	−0.057, 0.183	0.158	−0.022, 0.203	1.000	−0.060, 0.115

	**Left-side meningioma**		**Normal subject (*n* = 44)**						
	**PTBE- (*n* = 11)**	**PTBE+ (*n* = 25)**							

Right ALPS	1.272 ± 0.167	1.198 ± 0.204	1.215 ± 0.093	1.000	−0.079, 0.175	1.000	−0.075, 0.158	1.000	−0.095, 0.082
Left ALPS	1.513 ± 0.198	1.304 ± 0.178	1.273 ± 0.105	0.002	0.060, 0.317	<.001	0.110, 0.347	0.840	−0.050, 0.130

*Data are mean ± SD; CI, confidence interval; PTBE−, meningiomas without peritumoral brain edema; PTBE+, meningiomas with peritumoral brain edema.*

**FIGURE 3:**
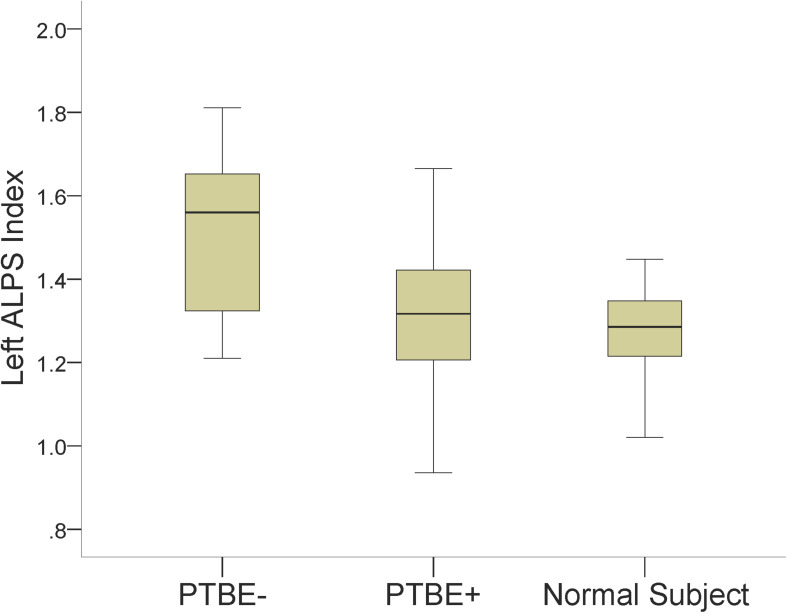
Boxplots show differences in the left ALPS index among left-side meningiomas without peritumoral brain edema (PTBE-), left-side meningiomas with peritumoral brain edema (PTBE+) and normal subjects.

The left ALPS index of left-side meningiomas without PTBE, left-side meningiomas with PTBE, and normal subjects was 1.513 ± 0.198, 1.304 ± 0.178, and 1.273 ± 0.105, respectively. The left ALPS index of meningiomas without PTBE was significantly higher than that of meningiomas with PTBE (*P* = 0.002) and normal subjects (*P* < 0.001). The left ALPS index was not significantly different between meningiomas with PTBE and normal subjects (*P* = 0.840). The contralateral (right) ALPS index of the three groups was not statistically significant (*P* = 0.625). Figure 3 shows the differences in left ALPS index among the three groups.

**FIGURE 4:**
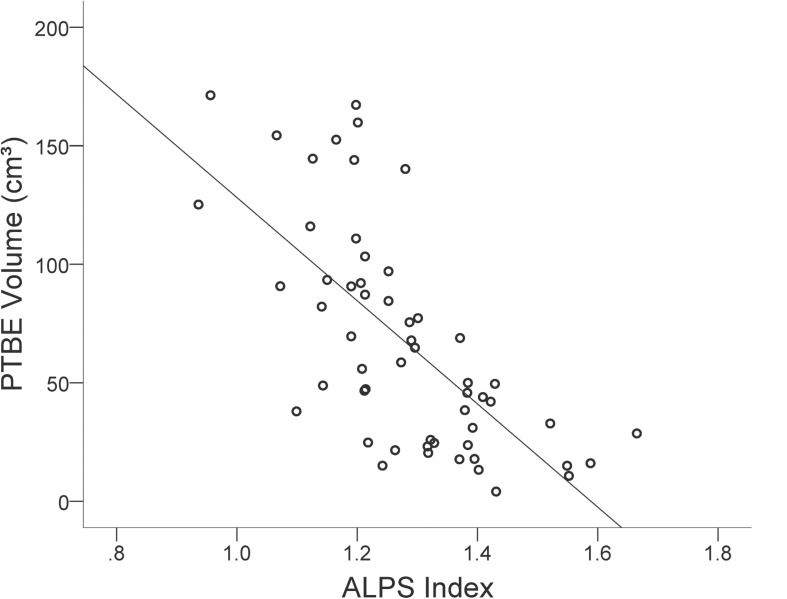
Scatterplot with regression line shows the negative correlation (*r* = –0.680, *P* < 0.001) between peritumoral brain edema (PTBE) volume (cm^3^) and ALPS index of meningiomas.

In the group of meningiomas with PTBE, volume of PTBE correlated inversely with ALPS index ipsilateral to tumor (*r* = −0.680, *P* < 0.001). No correlations were found between PTBE volume and age (*r* = −0.007, *P* = 0.956), sex (*r* = 0.165, *P* = 0.224), tumor volume (*r* = −0.167, *P* = 0.220), tumor location (*r* = 0.004, *P* = 0.976), tumor grade (*r* = 0.053, *P* = 0.696), or Ki-67 (*r* = 0.185, *P* = 0.337). Figure 4 shows the correlation between PTBE volume and ALPS index ipsilateral to meningiomas. On stepwise multiple linear regression analysis, ALPS index ipsilateral to tumor (β = −0.632; *P* < 0.001) was the only factor associated with PTBE volume. No correlations were found between PTBE volume and age (*P* = 0.226), sex (*P* = 0.856), tumor volume (*P* = 0.141), tumor location (*P* = 0.759), tumor grade (*P* = 0.713), or Ki-67 (*P* = 0.393).

## Discussion

In the present study, the ALPS index of meningiomas without PTBE was significantly higher than those of meningiomas with PTBE and normal subjects. Among meningiomas with PTBE, ALPS index is inversely correlated with PTBE volume. The relationship between PTBE volume and ALPS index of meningiomas suggested that PTBE formation may be related to glymphatic dysfunction.

The associations of PTBE extensions with many imaging, histologic and molecular features of meningioma have been reported. The brain parenchyma compression theory states that large meningiomas lead to compression, ischemia, and edema (Lobato et al., 1996). However, the associations between PTBE extension and tumor volume have been inconsistent. While some studies (Bitzer et al., 1997; Simis et al., 2008) reported positive correlations between PTBE extension and tumor volume, others found no correlations (Paek et al., 2002; Lee et al., 2008; Sapkota et al., 2020; Toh and Castillo, 2021). Some studies attributed frequently found and prominent PTBE in meningiomas with T2 hyperintensity to higher water content (Nakano et al., 2002; Lee et al., 2008; Kim et al., 2011; Sapkota et al., 2020). However, this explanation is not supported by a recent study (Toh and Castillo, 2021) which found no differences in tumor T2-weighted signal intensity and apparent diffusion coefficient between meningiomas without and with PTBE. In their study, the only factor associated with PTBE was tumor FA which reflects interstitial fluid flow directionality. Similar to a previous study (Gawlitza et al., 2017), our study shows no correlations between PTBE and histologic grade or Ki-67 index. Other studies reported correlations between PTBE and molecular features of meningioma including the expressions of vascular endothelial growth factor, aquaporin 4, and matrix metalloproteinase 9 (Gawlitza et al., 2017; Berhouma et al., 2019). Although some of these imaging, histologic and molecular findings of meningioma may be associated with PTBE, the underlying mechanisms for extra-axial tumor characteristics to cause intra-axial PTBE remain unclear. In our study, glymphatic function was the only significant factor associated with PTBE volume. To the best of our knowledge, this correlation has not been reported previously.

In the glymphatic pathway, a convective influx of CSF is balanced by the perivenous efflux of interstitial fluid (Iliff et al., 2012). The growth of meningiomas may disrupt this balance and result in the accumulation of interstitial fluid, i.e., PTBE. Compared to ALPS index of meningiomas with PTBE and normal subjects, the significantly higher ALPS index in meningiomas without PTBE may suggest increased glymphatic function that facilitates interstitial fluid clearance that reduces or prevents the formation of PTBE. In contrast, ALPS index was not significantly different between meningiomas with PTBE and normal subjects, indicating that no significant changes in glymphatic function for meningiomas with PTBE versus normal subjects. We herein speculate that changes in glymphatic function in response to meningioma growth may have an important role in PTBE formation. Insufficient glymphatic function for interstitial fluid clearance may result in PTBE formation.

As shown in previous studies, alterations in glymphatic function vary with diseases. Decreased glymphatic function and subsequently impaired waste clearance have been reported in Alzheimer’s disease, normal pressure hydrocephalus, small vessel disease, and traumatic brain injury (Nedergaard and Goldman, 2020). On the other hand, some clinical conditions are associated with increased glymphatic function. In acute ischemic infarction, edema appears to be caused by a rapid influx of CSF into the brain parenchyma (Mestre et al., 2020). In mice harboring gliomas and melanomas, glymphatic function is increased to reduce PTBE by remodeling of meningeal lymphatic vessels (MLVs) which is downstream of the glymphatic system (Hu et al., 2020). In mice with defective MLVs, impaired drainage of brain parenchymal interstitial fluid aggravates PTBE. Therefore, the increased glymphatic function observed in meningiomas without PTBE may be explained by tumor-induced remodeling of MLVs which facilitate efflux of interstitial fluid from the brain parenchyma. Alternatively, the higher ALPS index in meningiomas without PTBE suggests that this group of patients may have more glymphatic reserve capacity, which serves to relieve the PTBE.

In intra-axial tumors, tumor interstitium directly contacts the surrounding brain parenchyma. Their PTBE may be caused by disruption of the blood-brain barrier (Stummer, 2007) and vasogenic edema associated with tumor cell infiltration. In this setting, it would be difficult to determine the contribution of glymphatic dysfunction to PTBE formation. In contrast, meningioma is a good disease model to study the role of glymphatic system in PTBE formation since it is an extra-axial tumor, and in most cases, is separated from brain parenchyma by pia-arachnoid membrane. The relationship between PTBE volume and ALPS index of meningiomas observed in our study suggests that PTBE formation may be related to glymphatic dysfunction. To date, most studies of the human glymphatic system have focused on mapping its pathways (Ma et al., 2017, 2019a). To the best of our knowledge, no human studies have investigated tumor-related glymphatic dysfunction and thus, further studies are needed to confirm our observations.

MRI studies have confirmed the presence of glymphatic system in the human brain (Ma et al., 2017, 2019a). Intrathecal injection of gadolinium-based contrast agents allows simultaneous visualization of glymphatic system clearance, deep cervical lymph nodes, and putative meningeal lymphatic vessels (Zhou et al., 2020). However, this method is invasive and the off-label use of gadolinium-based contrast agents has potential neurotoxicity. Alternatively, several diffusion imaging-based techniques including DTI-ALPS (Taoka et al., 2017; Harrison et al., 2018; Debacker et al., 2020), have been used to investigate water movement along the perivascular spaces as a reflection of glymphatic system function. Besides being non-invasive, this approach measures glymphatic function quantitatively and reproducibly and allows investigating longitudinal changes of glymphatic function in the same subjects as well as comparison of glymphatic function across different patient groups.

There are limitations to our study. First, the glymphatic system has only recently been recognized and there are no well-established non-invasive methods to measure its function in humans. As fluid dynamics within each anatomical region are not yet fully understood, it is not known in which segments of glymphatic pathway should fluid flow be measured for an estimation of overall glymphatic function. Second, ALPS index evaluates water flow along deep medullary veins and it remains unclear if this measurement reflects the overall function of glymphatic system or just the efflux of interstitial fluid at the perivenous spaces. The correlation between water diffusivity in right-to-left direction along deep medullary veins and the glymphatic activity needs further validation. Third, our findings may not fully explain the pathogenesis of PTBE in meningiomas, but they may suggest a new approach to reduce PTBE, i.e., improving glymphatic function.

In conclusion, we found an inverse relationship between PTBE volume and ALPS index in meningiomas. PTBE formation in meningiomas may be related to dysfunction of the glymphatic system. Insufficient glymphatic function for interstitial fluid clearance during meningioma growth may explain PTBE formation.

## Data Availability Statement

Raw data supporting conclusions of this article are available from the corresponding author after signing an agreement.

## Ethics Statement

The studies involving human participants were reviewed and approved by the Chang Gung Medical Foundation Institutional Review Board. Written informed consent for participation was not required for this study in accordance with the national legislation and the institutional requirements.

## Author Contributions

CT, TS, and MC contributed to conception and design of the study. CT and TS contributed to acquisition and analysis of data. CT and MC contributed to drafting the text and preparation of figures. CT wrote the first draft of the manuscript. All authors read and approved the final manuscript.

## Conflict of Interest

The authors declare that the research was conducted in the absence of any commercial or financial relationships that could be construed as a potential conflict of interest.

## Footnotes

1 http://www.slicer.org
